# The Confidence Information Ontology: a step towards a standard for asserting confidence in annotations

**DOI:** 10.1093/database/bav043

**Published:** 2015-05-09

**Authors:** Frederic B. Bastian, Marcus C. Chibucos, Pascale Gaudet, Michelle Giglio, Gemma L. Holliday, Hong Huang, Suzanna E. Lewis, Anne Niknejad, Sandra Orchard, Sylvain Poux, Nives Skunca, Marc Robinson-Rechavi

**Affiliations:** ^1^Department of Ecology and Evolution, University of Lausanne, 1015 Lausanne, Switzerland, ^2^SIB Swiss Institute of Bioinformatics, 1015 Lausanne, Switzerland, ^3^Department of Microbiology and Immunology and Institute for Genome Sciences, University of Maryland School of Medicine, Baltimore MD, USA, ^4^SIB Swiss Institute of Bioinformatics, 1 Rue Michel Servet, 1211 Geneva, Switzerland, ^5^Department of Medicine and Institute for Genome Sciences, University of Maryland School of Medicine, Baltimore MD, USA, ^6^Department of Bioengineering and Therapeutic Sciences, University of California, San Francisco, CA 94158, USA, ^7^School of Information, University of South Florida, Tampa, FL, 33647, USA, ^8^Genomics Division, Lawrence Berkeley National Lab, 1 Cyclotron Rd., Berkeley, 94720 CA USA, ^9^European Molecular Biology Laboratory, European Bioinformatics Institute (EMBL-EBI), Wellcome Trust Genome Campus, Hinxton, Cambridge CB10 1SD, UK, ^10^Swiss-Prot Group, SIB Swiss Institute of Bioinformatics, Centre Medical Universitaire, Geneva, Switzerland, ^11^ETH Zurich, Department of Computer Science, Universitätstr. 19, 8092 Zürich, Switzerland, ^12^SIB Swiss Institute of Bioinformatics, Universitätstr. 6, 8092 Zürich, Switzerland and ^13^University College London, Gower St, London WC1E 6BT, UK

## Abstract

Biocuration has become a cornerstone for analyses in biology, and to meet needs, the amount of annotations has considerably grown in recent years. However, the reliability of these annotations varies; it has thus become necessary to be able to assess the confidence in annotations. Although several resources already provide confidence information about the annotations that they produce, a standard way of providing such information has yet to be defined. This lack of standardization undermines the propagation of knowledge across resources, as well as the credibility of results from high-throughput analyses. Seeded at a workshop during the Biocuration 2012 conference, a working group has been created to address this problem. We present here the elements that were identified as essential for assessing confidence in annotations, as well as a draft ontology—the Confidence Information Ontology—to illustrate how the problems identified could be addressed. We hope that this effort will provide a home for discussing this major issue among the biocuration community.

**Tracker URL:** https://github.com/BgeeDB/confidence-information-ontology

**Ontology URL:** https://raw.githubusercontent.com/BgeeDB/confidence-information-ontology/master/src/ontology/cio-simple.obo

## Introduction

Curation in biology has become essential for capturing information from publications or results from experiments, and for making these data available through public repositories. Whether to allow efficient data retrieval (e.g. functional annotations of single genes or gene products), or to make sense of the overwhelming amount of data produced by current technologies [e.g. gene ontology (GO) enrichment analyses on large datasets, or protein-protein interaction network analyses], this curation work provides us with standardized datasets that are essential for downstream analyses (1, 2). However, the curated data itself can often be difficult to assess, because it arises from different types of experiments and analyses, each with varied outputs at different levels of quality.

As the volume of biological data has grown, so has the amount of annotations available (3). This growth in turn creates a pressing need to assess the confidence in these annotations, to allow users to decide whether to use large sets of annotations with possible high rates of false positives, or more restricted sets of annotations of expected higher quality.

The type of evidence used to support the assignment of an annotation is often used as a proxy for judging its quality, in large part owing to the extensive use of the evidence ontology (ECO) (4). The ECO allows curators to provide information about the type of method used to support an annotation, for example experimental or computational. Due to the lack of a confidence evaluation system, the evidence terms have often been used as a proxy to evaluate the quality of certain data. However, evidence terms are not sufficient to infer confidence, and a same evidence term can be used to support annotations based on experiments of very different quality.

For example, ‘microarray evidence’ (ECO:0000058) may report results from a high quality experiment with several biological replicates, or from a single low quality experiment. Or a ‘protein BLAST evidence’ (ECO:0000208) could correspond to a weak similarity over part of the protein, or 99% identity over the whole length of the protein. Another example is the use of annotations automatically assigned by computational methods, without curator supervision, tagged with the related evidence term (ECO:0000501 or GO evidence code IEA); they have often been considered the least reliable, whereas after evaluation, these annotations appeared to be as reliable as curated non-experimental annotations (1).

Although evidence sources and quality of annotations are intertwined, they are nevertheless two different concepts, and users would be better served if they were captured separately.

Several groups have implemented methods for addressing the problem of heterogeneous quality of annotations that are derived from the same source, and for estimating the confidence in the annotations they provide. For instance, the ChEMBL team has defined a confidence score ranging from 1 to 9, assessing both the quality of protein targets, and of the curation process (5); the Bgee team has been using a controlled vocabulary to assess confidence in homology relations between species-specific anatomical structures, ranging from ‘uncertain’ to ‘well-established’, depending on the agreement level found in literature (6); neXtProt (7) classifies data and annotations with ‘gold’, ‘silver’ and ‘bronze’ qualifiers to represent data quality; and UniProtKB/Swiss-Prot (8) provides an annotation score ranging from 1 to 5 at the level of the protein entry, which documents both the quantity of annotations and their provenance.

Several resources also provide distinct datasets of different qualities. For example, UniProtKB/Swiss-Prot distinguishes ‘unreviewed’ from ‘reviewed’ entries, the latter consisting of manually curated records providing a critical review of experimental data from literature, as well as curator-evaluated computational analysis; such a distinction is also used by the Catalytic Site Atlas (9), and MACiE (10); similarly, the NCBI provides RefSeq (11), a subset of better annotated sequences.

These different datasets represent an attempt to address the problem of annotation confidence assessment, and have proven their usefulness. For example, because the high-quality datasets are expected to have a lower false positive rate, they often serve as templates for transitively assigned annotations, e.g. to define protein names for bacterial genome annotations (12).

As useful as they are, the above efforts lack standardization. Although the precise means of assessing confidence is highly dependent on the data type (13), it should be possible to define a standard way to encode and provide this information. To that aim, a workshop was organized during the Biocuration 2012 Conference (14), followed by discussions though a wiki and an un-conference at Biocuration 2014. The work described here is a community effort, resulting from discussions between several groups who wished to capture statements of confidence information about annotation assertions in a more systematic manner, i.e. statements of confidence information about assertions.

This article presents the consensual principles that arose from these discussions, as well as a draft ontology used as a proof of principle: the Confidence Information Ontology (CIO). Since the CIO is still in relatively early stages of development, we anticipate that there might be significant changes to the terms, as the community begins to explore the use of CIO. With this publication, we hope to expand the set of active CIO developers and users, so as to build an optimal ontology for this domain. We then provide suggestions of implementation, to highlight how the use of such an ontology could address the problem of standardizing confidence information about annotations.

### Relationship between evidence, quality and confidence

Annotations are created on the basis of available evidence lines. In this work, evidence is considered to be any scientific evidence obtained from laboratory experiments, computational methods, or manual curation, as described in the ECO. Confidence in a particular assertion can then be defined, based on the set of related evidence lines.

Although confidence and quality are related concepts, they are nevertheless distinct. Quality refers to the value of the source or of the annotation, whereas confidence refers to the level of certainty that an assertion is correct. For instance, several evidence lines of low quality, produced by methods known to be noisy (e.g. yeast two-hybrid), could yield an assertion of high confidence, because they are repeatedly confirmed (e.g. with different reporter genes); or, an annotation could be of poor quality because of missing information, but capture a high confidence assertion.

In this manuscript, we aim at defining how to provide global confidence about annotations, and not at defining criteria to assess quality of evidence.

### Standardizing confidence information

To convey confidence information in a standard way, an obvious solution is to use an ontology, to provide standard terms for assessing confidence, in the same way the ECO provides standard terms to annotate evidence types. Although it is neither feasible nor desirable for different annotation groups to use a single definition of quality metrics, it should be feasible and desirable for each group to map their quality assessments to terms from a common ontology.

**Figure 1.**
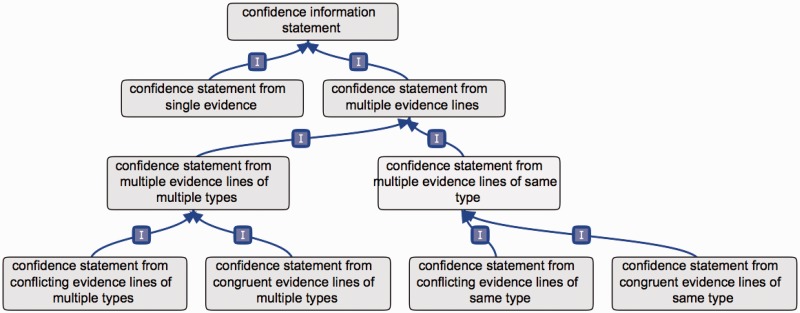
Partial overview of the CIO. The first branching of the CIO distinguishes annotations supported by a single evidence, or by multiple evidence lines. In the latter case, further subclasses refine the overall confidence in the annotation, yielded from all evidence lines available considered together.

In order to demonstrate how such an ontology could address the need for a standard method to provide confidence information, we present here a new ontology that we have developed: the CIO (Figure 1). This ontology was created to address the main points identified to provide clear and meaningful information about confidence in annotations. Here, we present the rationale behind the design of this ontology, followed by suggestions for implementing its use, which also highlight the issues it addresses. The ontology implementation is described in the supplementary material.

### Asserting confidence in single evidence

Parameters for determining confidence in annotations are highly heterogeneous among groups working on different data types. Yet it is possible to summarize the confidence information using a basic rating system. Each group working on a specific data type could define clear parameters to assign these levels of confidence, and several groups have already implemented such a rating system (see Introduction).

**Figure 2.**
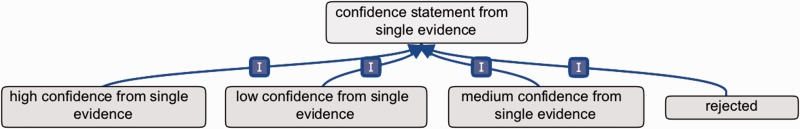
Overview of the confidence statement from single evidence branch. The CIO defines three basic confidence statements, corresponding to a simple rating system, that can be modularly used for single evidence annotation, plus a rejected term, used to tag retracted results.

Three CIO terms corresponding to basic levels of confidence have been defined in the ontology: ‘low confidence from single evidence, medium confidence from single evidence, and high confidence from single evidence’ (Figure 2). These confidence statements might summarize different quantitative measures, e.g. numeric scores, minimum information requirements or standards for experimental procedures. Computational annotations could also be given a confidence score, based, e.g. on an E-value or *P*-value threshold, or a percentage of identity.

An additional term, ‘rejected’, allows to tag assertions that were retracted, for instance, following paper retraction, author misinterpretation or curator misinterpretation. This term is used to circumvent the fact that, when results are retracted, associated annotations are often deleted. Consequently, end-users might not be aware that a result was annotated, and then shown to be incorrect. Annotating an assertion with this confidence term would allow to keep this information available, and keep track of the invalidation. Note that this is different from negative annotations, used to negate annotation interpretation. It is also different from conflicting multiple evidence lines, where each single evidence has not been directly invalidated. Rejection is a stronger assertion about an annotation source than conflict, capturing that this evidence should no longer be used.

These basic confidence statements should be used for annotating each individual evidence. While it might seem an added burden to annotate an assertion with yet another ontology term, we believe that curators already informally assess evidence sources they use. Thus in many cases a basic rating system could be applied with little added effort (see Discussion).

Although some evidence types can have numerical estimates of confidence (e.g. BLAST score), others can only be judged qualitatively (e.g. non-traceable author statement in GO). Confidence information should always be examined in the light of the evidence source used. For this reason, we believe that annotations using the CIO should always be provided along with terms from the ECO. We also believe that for those evidence sources that allow numerical estimates, annotation providers should always publish the parameters used to assign a confidence statement.

We are hopeful that, in the long run, agreement on these parameters might be reached by different groups working in the same field. For example, it might be possible to define a certain level of sequence identity that would be a good predictor for a particular class of GO terms; or, certain experimental setup that would provide more trustworthy protein-protein interaction data. It is the standardization of annotation assignment in ontologies such as ECO and CIO that facilitates this.

### Global confidence from aggregation of multiple evidence lines

The strongest point of agreement among all workshop participants was that assertions supported by multiple evidence lines are more reliable than assertions supported by a single line of evidence. This means that a global confidence in an assertion can arise from taking all available evidence into account. This led us to create two branches in the ontology: a ‘confidence statement from single evidence’ branch’ and a ‘confidence statement from multiple evidence lines’ branch (Figure 1). The former term is the parent of the basic single-evidence terms described above (Figure 2).

The best practice would be that for assertions supported by multiple evidence lines, each individual evidence would still be assigned a term from the ‘confidence statement from single evidence’ branch. The overall confidence in the assertion, assessed from all available evidence lines, would then be summarized using a term from the ‘confidence statement from multiple evidence lines’ branch.

### Global confidence from multiple experimental or computational types

Another consensual point was that different, non redundant, experimental or computational methods provide a stronger support for an annotation. For this reason, we created the terms ‘confidence statement from multiple evidence lines of same type’ and ‘confidence statement from multiple evidence lines of multiple types’ (Figure 1), where evidence type corresponds to any evidence term in ECO.

An example of the stronger support provided by evidence lines of multiple types can be found in the annotations of homology between anatomical entities, provided by the Bgee team (see https://github.com/BgeeDB/anatomical-similarity-annotations/wiki/Similarity-annotations). For instance, an assertion states that the urinary bladder is homologous among *Tetrapoda*. Some evidence lines provided (15, 16) are from the type ‘phylogenetic distribution evidence’ (as the structure is present in various *Tetrapoda* species). These individual statements are of medium confidence, and if we were to integrate these two lines of evidence of the same type, the confidence would be unchanged. However, the assertion is also supported by an evidence of type ‘developmental similarity evidence’ (16), which allows to corroborate the assertion, using a different evidence type, and to grant the overall annotation high confidence.

A similar procedure is applied by curators of the International Molecular Exchange (IMEx) consortium in the field of protein interactions (17), with the aim of capturing all of the experimental details available.

Such a summary annotation, taking all evidence lines into account, could be automatically produced, with rules specifying how individual confidence values should contribute to the overall confidence (see section Suggestions of implementation for an example). It would also be possible to decide to not formally assess confidence for each individual evidence, but to manually annotate only the overall assertion, which would represent an advantage for curation teams with limited resources.

### Reconciling congruent and conflicting evidence lines

Finally, the information provided by multiple evidence lines needs to be reconciled. When all evidence lines supporting an assertion are congruent, the assertion should be trusted with higher confidence than when some evidence lines are conflicting. Congruent assertions could be, for instance, annotations between the same gene product and GO term, based on different evidence sources. Conflicting assertions are annotations yielding opposite interpretation, for instance, a positive annotation between a gene product and a GO term, and a negative annotation between the same gene product and GO term, using the ‘NOT’ qualifier; or incompatible GO terms assigned to the same gene product, for instance the GO term ‘DNA replication’ from the ‘Biological Process’ branch, and the GO term ‘cytoplasm’ from the ‘Cellular Component’ branch; in any case, it is the resources implementing the CIO that should define what a conflict is, for the type of data that they annotate.

When evidence lines are contradictory, we can distinguish ‘weak’ contradictions (e.g. a single low-confidence evidence contradicting several high-confidence evidence lines), from ‘strong’ contradictions (e.g. several high-quality evidence lines, all contradictory).

**Figure 3.**
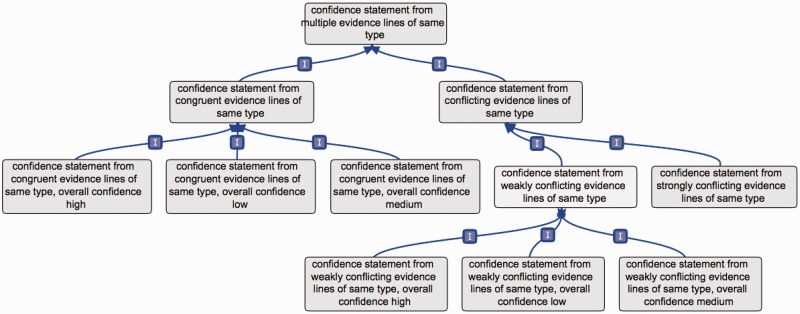
Example of conflicting versus congruent terms. This figure presents the branch ‘confidence statement from multiple evidence lines of same type’; the rationale would be the same if applied to evidence lines of multiple types. The term confidence statement from multiple evidence lines of same type has two subclasses: ‘confidence statement from conflicting evidence lines of same types’ and ‘confidence statement from congruent evidence lines of same type’. The ‘congruent evidence lines’ term has three subclasses, to define the overall level of confidence obtained from the set of supporting evidence lines. Similarly, the ‘weakly conflicting evidence lines’ term has three subclasses, defining the overall level of confidence obtained from the set of available evidence lines. The ‘strongly conflicting evidence lines’ term does not have such subclasses, as in that case, the evidence lines do not allow to reach a consensual conclusion.

For these reasons, the ‘same type’ and ‘multiple types’ terms each have two subclasses, e.g. when evidence lines are of the same type: ‘confidence statement from congruent evidence lines of same type’ and ‘confidence statement from conflicting evidence lines of same type’. Such a ‘conflicting evidence lines’ term also has two subclasses, e.g.: ‘confidence statement from strongly conflicting evidence lines of same type’ and ‘confidence statement from weakly conflicting evidence lines of same type’ (Figure 3).

The term ‘confidence statement from congruent evidence lines of same type’ has three subclasses, based on the overall confidence obtained from the associated evidence lines (Figure 3). For instance, if an annotation is supported by two evidence lines, a ‘high confidence assertion’ and a ‘medium confidence assertion’, the best confidence is ‘high confidence assertion’, and the term used for the overall annotation could then be: ‘confidence statement from congruent evidence lines of same type, overall confidence “high”’. Or, if an assertion is repeatedly confirmed by many low-confidence individual evidence lines, the overall confidence obtained could still be high. It is the responsibility of the annotation teams to define and communicate the parameters, relevant to their field, which are used to produce overall confidence.

The term ‘confidence statement from weakly conflicting evidence lines of same type’ has similar subclasses. Indeed, when using weakly contradicting evidence lines, the implication is that the overall assertion is believed to be true. It is then possible to provide an overall confidence from the set of available evidence lines. The following three subclasses were thus created: ‘confidence statement from weakly conflicting evidence lines of same type, overall confidence “high”’; ‘confidence statement from weakly conflicting evidence lines of same type, overall confidence “medium”’; ‘confidence statement from weakly conflicting evidence lines of same type, overall confidence “low”’.

Such subclasses were not deemed relevant for the term ‘confidence statement from strongly conflicting evidence lines of same type’. In that case, the supported assertion is believed to need further validation, as evidence lines yield different conclusions of similar confidence. Assertions tagged with this term should be targeted in priority when integrating new findings, as they are more likely to evolve, following the development of new methods or technologies. The individual single-evidence confidence annotations associated to strongly conflicting evidence lines would be in that case useful to define directions for further analyses.

Note that the rationales are the same when using terms from the branch ‘confidence statement from multiple evidence lines of multiple types’. Also, assertions annotated with the term ‘rejected’ should not be considered when aggregating multiple evidence lines.

### Example of a potential workflow using GO annotations

An example of use would be to extend the GO annotation conventions (http://geneontology.org/page/go-annotation-conventions). Please note that this is an illustrative example, and that the implementation of such conventions is pending further discussion within the GO consortium. We provide this example only to present how the CIO could be used. For an example of an actual implementation, see next section.

The current recommendations of the GO consortium are that each annotation must include a gene product identifier, a GO identifier, and an evidence term identifier. If multiple sources support an annotation, then multiple annotations with identical GO identifiers and reference identifiers, but different evidence terms, may be made.

A first possible modification could be to additionally annotate each of these individual annotations with a term from the branch ‘confidence statement from single evidence’; this would require defining clear parameters to assign confidence for each evidence type.

For instance, in the GO annotation file gene_association.goa_uniprot_noiea (retrieved as of 3 February 2015, see http://geneontology.org/page/download-annotations), the product of the *Candida* *glabrata* gene ERG9 (UniProtKB ID Q9HGZ6) is associated to the GO term GO:0051996 ‘squalene synthase activity’, with two annotations:

- One of them is supported by the evidence term ECO:0000200 ‘sequence alignment evidence’ (GO evidence code ISA). It was produced based on a publication (18) providing an alignment of the deduced amino acid sequence of this protein to Erg9p sequences in five other species. The alignment was performed on a predicted open reading frame of 443 amino acids, and exhibited from 33.4% identity in *Arabidopsis* *thaliana*, to 71.3% identity in *Saccharomyces cerevisiae*, and identified three conserved predicted kinase motifs. This protein sequence is thus highly conserved on a significant length, even in distant species, with relevant motifs found; this annotation could be assigned the term CIO:0000003 ‘high confidence from single evidence’. Of note, the GO consortium does not define numerical cutoffs for the extent or percentage identity of sequence similarity (see http://geneontology.org/page/isa-inferred-sequence-alignment). These cutoffs should vary, depending on, e.g. the organism studied, or the protein function captured. Again, each annotation team should define and communicate clear parameters to assign confidence.
- The other annotation is supported by the evidence term ECO:0000015 ‘mutant phenotype evidence’ (GO evidence code IMP), and was produced on the basis of the same publication. The authors generated *C. glabrata* strains where ERG9 was under the control of the tetracycline-regulatable promoter. The correct replacement of the endogenous ERG9 promoter was verified by PCR, and the ERG9-controllable strains exhibited a severe growth defect in medium with DOX; the growth defect caused by DOX was suppressed by the addition of serum containing exogenous cholesterol. Overall, as this experiment was carefully designed, and provided a clear result, this annotation could also be assigned the term CIO:0000003 ‘high confidence from single evidence’.

Another potential modification could be to generate an additional summary line, when several annotations are available in support of an assertion, summarizing all of them; this additional line could be assigned a term from the ‘confidence statement from multiple evidence lines’ branch. This approach would have several advantages, and notably would provide a clear overview to end-users about the status of an association.

In the example described above, as the evidence lines provided are of different types, and are all of high confidence, the summary annotation could be assigned the term CIO:0000012 ‘confidence statement from congruent evidence lines of multiple types, overall confidence high’. This represents the highest level of confidence from the CIO. Of interest, it is possible to automatically determine whether two evidence lines are of a same or of different types, using the structure of the ECO.

To retrieve the evidence terms and references used to produce the additional summary line, either the corresponding single-evidence annotation lines (notably with the same gene product identifier and GO identifier) could be used if provided, or the multiple evidence terms and references could be integrated in the summary line as a list (as it is already possible for, e.g. columns 6 and 11 of the GO Annotation File, albeit for a different purpose, see http://geneontology.org/page/go-annotation-file-gaf-format-20). The summary line and the summary confidence term could also be automatically produced from the set of individual assertions, as it is already the case in the Bgee project (see next section).

In the GO annotation file format, it is also possible to provide negative assertions, using the NOT qualifier (column 4, see http://geneontology.org/page/go-annotation-file-gaf-format-20). Such negative assertions can be in conflict with other, positive, assertions, a conflict that could be captured in the summary line, with the use of terms from the branches ‘confidence statement from conflicting evidence lines of same type’ and ‘confidence statement from conflicting evidence lines of multiple types’. This would allow a simple and clear overview of the status of a particular assertion.

### Example of implemented workflow to annotate anatomical homology

An example of use can be found in the annotation of homology between anatomical entities, provided by the Bgee team (see https://github.com/BgeeDB/anatomical-similarity-annotations/wiki/Similarity-annotations). The format of this annotation file is inspired from the GO annotation file format (see http://www.geneontology.org/GO.format.gaf-2_0.shtml), and the procedure to provide supporting information is inspired from the guide to GO evidence codes (see http://www.geneontology.org/GO.evidence.shtml); additionally, the procedure follows the guidelines described here.

For example, to annotate the homology of the autopod among *Vertebrata*, there exist two alternative hypotheses: one considering that the autopod was a novel feature of *Tetrapoda*, another one considering that it appeared earlier, during *Vertebrata* evolution (19). These hypotheses notably allow to produce two annotations, positive and negative, about this homology originating in *Vertebrata*. Each of these assertions is manually annotated in Bgee, notably with evidence and confidence terms. Because these assertions represent general accepted knowledge in the field, but were captured only through the term ECO:0000033 ‘traceable author statement’, they were each assigned a medium confidence (annotations retrieved as of February 3 2015).

An overall assertion, taking into account each line of evidence, is then automatically produced, summarizing whether the evidence lines are conflicting or congruent, and using the ECO to decide whether they are of a same or different types (Table 1). As the two evidence lines are of a same confidence, the overall assertion is assigned a ‘strongly conflicting’ confidence term.

**Table 1.** Example homology annotation from Bgee

Entity name	Qualifier	Taxon name	Line type	Evidence term name	Confidence term name	Reference ID
Autopod	—	*Vertebrata*	RAW	Traceable author statement	Medium confidence from single evidence	PMID:23598338
Autopod	NOT	*Vertebrata*	RAW	Traceable author statement	Medium confidence from single evidence	PMID:23598338
Autopod	—	*Vertebrata*	SUMMARY		Confidence statement from strongly conflicting evidence lines of same type	—

This table shows columns 4, 5, 7, 8, 10, 12 and 13 of the Bgee homology annotation file. The first two rows represent conflicting annotations from single evidence, about the homology of the autopod among *Vertebrata*. The third is an auto-generated row, summarizing the status of this homology hypothesis, from all evidence lines available.

Finally, the terms from the CIO that are the most informative and likely to be used are described in Table 2.

**Table 2.** List of most informative terms from the CIO

Interpretation	Main branch	Term label
Assertion should be trusted	Single evidence	High confidence from single evidence
	Multiple evidence lines, same type	Confidence statement from congruent evidence lines of same type, overall confidence high
		Confidence statement from congruent evidence lines of same type, overall confidence medium
	Multiple evidence lines, multiple types	Confidence statement from congruent evidence lines of multiple types, overall confidence high
		Confidence statement from congruent evidence lines of multiple types, overall confidence medium

Assertion needs additional support	Single evidence	Low confidence from single evidence
	Multiple evidence lines, same type	Confidence statement from strongly conflicting evidence lines of same type
		Confidence statement from weakly conflicting evidence lines of same type, overall confidence low
	Multiple evidence lines, multiple types	Confidence statement from strongly conflicting evidence lines of multiple types
		Confidence statement from weakly conflicting evidence lines of multiple types, overall confidence low

## Discussion

The aim of this work is to show how confidence in annotations can be provided in a standard way; it is not to impose one specific practice in assessing confidence. One purpose of the draft CIO described in this manuscript is to invite feedback and comments from the community. Whatever solution is eventually adopted, the problem of assessing confidence in annotations must be addressed. We hope that this project will provide a home for discussing this major issue (ideally through its associated tracker, available at https://github.com/BgeeDB/confidence-information-ontology), as well as a practical solution for those who wish to rapidly implement it. Once the principle and design of the CIO are approved by the community, the formalization of this draft ontology could then be improved, by properly defining the semantics of the terms created, using, e.g. the Ontology for Biomedical Investigations (20), or the Information Artifact Ontology (https://code.google.com/p/information-artifact-ontology/).

The main practical additional task described here is annotating single-evidence assertions with a confidence statement using a basic rating system, a solution akin to what is already adopted by several resources (see Introduction). Summary confidence annotations could then be automatically generated and assigned a confidence term from the ‘confidence statement from multiple evidence lines’ branch, as it is the case, e.g. in the Bgee homology annotations, as long as individual assertions are provided with confidence information and ECO terms. Alternatively, annotation teams with limited resources could choose to provide annotations only at the global summary level. However, the latter solution has the disadvantage of masking the evaluation of confidence at the level of each evidence, which limits the transparency of annotations.

Even when it is impossible to provide confidence in each statement, whether because of lack of manpower or because of methodological limitations (e.g. in case of many electronic annotation methods), it is still very relevant to record whether one evidence line or several supported an assertion, whether they were of the same type or not, and whether they were contradictory or consistent. Such an approach can also be used to integrate the CIO with legacy annotations, by imposing an arbitrary confidence statement to all single evidence lines (e.g. medium confidence from single evidence), and then automatically generating terms from the multiple evidence lines branch, thus providing confidence information at least according to the multiplicity and consistency of evidence lines.

Assessing the quality of the data being captured is one of the most difficult, yet essential, tasks of biocurators. Until now, this has not been done systematically, or even explicitly. There are several reasons for this: first, all papers go through a process of peer-review, and published data is usually assumed to be trustworthy. Second, due to the scale of the task, the resources available are not sufficient to capture the data from all published papers; a selection must be done as to which papers provide the most relevant information for the users of the resource being developed. As curated databases’ usage increases, biocurators have an editorial role that effectively filters published articles into biological databases. A careful selection of the data is thus essential.

This opens the question of defining what makes a ‘high confidence’ evidence. Many biocurators are accustomed to estimating the confidence in evidence sources that they use, yet it can be difficult to transform such subjective estimates into standardized levels. This issue is akin to inter-curator agreement for GO annotations (21), that despite being highly consistent, highlights the inevitable subjectivity of the process of assigning an ontology term to an assertion.

Indeed, biocuration is a translation problem: the language of biologists must be translated into a structured vocabulary suitable for computational analyses. Ideally, it would be done without losing any of the original meaning, but that is hardly possible. An important aspect which is often missing from annotations is their biological context. For example, a protein may be found in the nucleus in one article by some immunocytochemistry approach, but may be known to have a function that is more consistent with a mitochondrial localization. Ideally at some point, one should be able to integrate all information and try to reconstruct the biological meaning of the annotations. Having a confidence in the different annotations that describe a proteins’ role will certainly help to resolve some of the apparent discrepancies in the annotations (and in the literature).

One important feature we are proposing is to systematically provide a summary annotation when several evidence sources are available. We believe that the use of a summary annotation would be of great benefit, by allowing to have a clear overview of an assertion, taking into account all evidence lines. This can often be difficult when many sources are available, especially when they are contradictory.

Another advantage of the guidelines proposed here is the ability of maintaining erroneous assertions, for informing users about retracted results, while being able to discard them to produce summary interpretations. Indeed, while resources providing a global overview of annotations about an entry, such as UniProtKB/Swiss-Prot, can remove erroneous information, and provide comments to warn about incorrect information, this is hardly possible for resources presenting data based on individual assertions. For instance, for GO annotations, while the presence of the NOT qualifier allows to track conflicting information, it is not sufficient since information from dubious publications remains. Moreover, when an annotation is removed, e.g. following a paper retraction, no trace of this annotation is maintained. A user coming across the original article, unaware of the retraction, might conclude that the publication has just not yet been annotated.

An example of this issue was described by Poux *et al*. (22), who showed how erroneous statements about the SIRT5 protein, based on incomplete *in vitro* studies, are repeatedly published, still today. The approach proposed here would allow to identify assertions that have later been shown to be based on misleading conclusions, owing to the use of the ‘rejected’ term. Users would then be aware of the retraction, while retracted results would not impact summary annotations, presenting the correct interpretation. Also, as summary annotations can be generated automatically (as long as individual assertions are provided with confidence information and ECO terms), the reevaluation of an incorrect statement could be easily propagated to the summary annotation.

An aspect that is not addressed by the guidelines provided here is the different levels at which confidence in assertions can be estimated: at the level of the experimental procedure; at the level of the author interpretation, as authors might have selected results not allowing an unbiased interpretation; and at the level of the annotator interpretation. For now, the basic terms from the ‘confidence statement from single evidence’ branch should be used to take into account these different layers all together. The CIO could be expanded if that turned out to be a need of the community. Possible solutions could be to capture the confidence at these different levels independently, or to modify the branch ‘confidence statement from single evidence’ for this purpose. We hope that the current work will promote discussions towards this aim, possibly through the associated tracker.

This issue is related to the definition of the provenance of an annotation. Data provenance aims at documenting origin of data, but also annotation steps or task workflow (see http://www.w3.org/TR/prov-overview/). However, in the current state, documenting the full provenance of annotations might be overwhelming for most curation teams. The CIO is designed to be easily used in daily curation work, and to be as close as possible to the rating systems already adopted by several resources. Capturing provenance of an annotation in accordance with W3C and other standards (e.g. linked data frameworks) should be a long term goal; our current work represents a first step towards such structured capture.

We believe that the CIO, as well as the guidelines presented here, will allow end-users to better evaluate the pertinence of curated data. This is expected to enhance data dissemination across resources, as well as analyses based on curated data, thanks to the improved possibilities of filtering data, and of evaluating their trustworthiness.

## Conclusion

With the growth of annotations available, it has become essential to assess confidence in these annotations. This article is an attempt at defining guidelines for standardizing the exchange of confidence information, and at showing the feasibility of this approach.

We propose three basic principles: (i) while it is difficult to standardize parameters to define confidence in annotations across resources, it is possible to use a common ontology language to provide this information; (ii) in the same way that the GO guidelines recommend to provide annotations at the level of each individual evidence, a confidence statement might also be assigned to each single evidence, using a basic rating system; (iii) when several evidence lines are available relative to an assertion, it is desirable to provide a global summary assertion, taking all evidence into account.

We created the CIO in order to illustrate these principles. We hope it might be a trigger for the biocuration community to address this need for standardizing confidence information. Whether this ontology undergoes major changes in the near future, following feedback from the community, or whether it is used ‘as is’ by several resources, we hope that annotation confidence will be increasingly available in biocuration efforts.

## Supplementary Data

Supplementary data are available at *Database* Online.
